# Identification of Novel Susceptibility Loci for Kawasaki Disease in a Han Chinese Population by a Genome-Wide Association Study

**DOI:** 10.1371/journal.pone.0016853

**Published:** 2011-02-04

**Authors:** Fuu-Jen Tsai, Yi-Ching Lee, Jeng-Sheng Chang, Li-Min Huang, Fu-Yuan Huang, Nan-Chang Chiu, Ming-Ren Chen, Hsin Chi, Yann-Jinn Lee, Li-Ching Chang, Yi-Min Liu, Hsiang-Hua Wang, Chien-Hsiun Chen, Yuan-Tsong Chen, Jer-Yuarn Wu

**Affiliations:** 1 School of Chinese Medicine, China Medical University, Taichung, Taiwan; 2 Department of Medical Genetics, Medical Research and Pediatrics, China Medical University Hospital, Taichung, Taiwan; 3 Department of Health and Nutrition Biotechnology, Asia University, Taichung, Taiwan; 4 Institute of Biomedical Sciences, Academia Sinica, Taipei, Taiwan; 5 National Genotyping Center, Academia Sinica, Taipei, Taiwan; 6 Graduate Institute of Integrated Medicine, China Medical University, Taichung, Taiwan; 7 Divisions of Cardiology, Department of Pediatrics, China Medical University and Hospital, Taichung, Taiwan; 8 Department of Pediatrics, National Taiwan University Hospital, College of Medicine, National Taiwan University, Taipei, Taiwan; 9 Department of Pediatrics, Mackay Memorial Hospital, Taipei, Taiwan; 10 Graduate Institute of Chinese Medical Science, China Medical University, Taichung, Taiwan; 11 Department of Pediatrics, Duke University Medical Center, Durham, North Carolina, United States of America; 12 Graduate Institute of Chinese Medical Science, China Medical University, Taichung, Taiwan; Murdoch Childrens Research Institute, Australia

## Abstract

Kawasaki disease (KD) is an acute systemic vasculitis syndrome that primarily affects infants and young children. Its etiology is unknown; however, epidemiological findings suggest that genetic predisposition underlies disease susceptibility. Taiwan has the third-highest incidence of KD in the world, after Japan and Korea. To investigate novel mechanisms that might predispose individuals to KD, we conducted a genome-wide association study (GWAS) in 250 KD patients and 446 controls in a Han Chinese population residing in Taiwan, and further validated our findings in an independent Han Chinese cohort of 208 cases and 366 controls. The most strongly associated single-nucleotide polymorphisms (SNPs) detected in the joint analysis corresponded to three novel loci. Among these KD-associated SNPs three were close to the *COPB2* (coatomer protein complex beta-2 subunit) gene: rs1873668 (*p* = 9.52×10^−5^), rs4243399 (*p* = 9.93×10^−5^), and rs16849083 (*p* = 9.93×10^−5^). We also identified a SNP in the intronic region of the *ERAP1* (endoplasmic reticulum amino peptidase 1) gene (rs149481, *p_best_* = 4.61×10^−5^). Six SNPs (rs17113284, rs8005468, rs10129255, rs2007467, rs10150241, and rs12590667) clustered in an area containing immunoglobulin heavy chain variable regions genes, with *p_best_*-values between 2.08×10^−5^ and 8.93×10^−6^, were also identified. This is the first KD GWAS performed in a Han Chinese population. The novel KD candidates we identified have been implicated in T cell receptor signaling, regulation of proinflammatory cytokines, as well as antibody-mediated immune responses. These findings may lead to a better understanding of the underlying molecular pathogenesis of KD.

## Introduction

Kawasaki disease (KD) is the commonest cause of acquired heart disease among children in developed/industrialized countries. The incidence of KD varies between different countries, with Asian countries having a higher incidences than Western countries. Japan has the highest annual incidence in the world with 200 KD cases per 100,000 children under age 5 [1], [2], followed by Korea, with an annual incidence of 113.1 KD cases per 100,000 children under 5 years of age [3]. The incidence in Taiwan is 69 KD cases per 100,000 children under age 5 [4], [5], which ranks third after Japan and Korea. KD is an acute, self-limiting vasculitis of infants and children. Symptoms include prolonged fever that is unresponsive to antibiotics, polymorphous skin rash, swollen glands, red eyes, inflammation of the mouth, extensive rash, and swollen and red hands and feet. Characteristic peeling of the skin on fingers and toes occurs during convalescence [6]. Coronary aneurysms develop in 15%–25% of untreated children [6], [7]. The cause of this disease is currently unknown, although clinical and epidemiological features strongly suggest that an infectious agent triggers KD and genetic predisposition may underlie its etiology. It has been proposed that an inflammatory stimulus sets in motion a cascade of events that leads to host immune dysregulation in genetically predisposed individuals. During the acute stage of KD, infiltration of CD8-positive T cells and macrophages, activation of vascular endothelial cells, and increased serum levels of proinflammatory cytokines lead to inflammation and injury of blood vessels [8], [9]. Thus, identification of predisposing genetic factors would greatly facilitate understanding of the disease etiology and pathophysiology.

A number of genes that participate in immune-regulatory responses as well as cardiovascular-related loci have been extensively studied for an association with KD susceptibility or disease outcome using the candidate gene approach [10]. Several candidate genes have been examined in independent cohorts of the same or different ethnicity; however, the results are often conflicting. Most previous studies have been carried out on a single small cohort of KD patients, and findings were reported without validation in additional case-control sets. There have been few genome-wide studies of this disease. A genome-wide linkage analysis conducted with Japanese KD sib-pair samples revealed several linked regions, and led to the identification of a functional polymorphism in the *ITPKC* (inositol 1,4,5-triphosphate 3-kinase C) gene [11], [12]. A genome-wide association study (GWAS) conducted in an international cohort of Caucasian patients identified variants of genes with functions potentially related to inflammation, apoptosis, and cardiovascular pathology with *NAALADL2* (rs17531088, *p*_combined_ = 1.13×10^-6^ and *ZFHX3* (rs7199343, *p*_combined_ = 2.37×10^-6^ most significantly associated [13].

To identify novel mechanisms that might predispose patients to KD, we conducted a two-stage GWAS scan for KD in a Han Chinese population residing in Taiwan. In this paper, we present results of the first GWAS for KD in a Han-Chinese population, and identify new KD susceptibility loci that are involved in the immune response and are associated with increased risk of KD.

## Results

### Data quality

Individual call rates were greater than 0.95 for all subjects genotyped in this study. The average individual call rate was 0.993 (standard deviation [SD]  = 0.005). Of the 906,955 SNPs on the Affymetrix 6.0 SNP chip, 96,955 were non-polymorphic in both KD cases and controls; 27,779 had a call rate of <95%; 30,080 had an overall minor allele frequency (MAF) <5% and a total call rate <99%; and 28,093 exhibited a significant deviation from Hardy–Weinberg equilibrium compared to the control group (*p*<10^−7^). A total of 723,638 SNPs (79.8%) that passed a quality control filter with an average SNP call rate of 0.996 (SD = 0.006) were included in the GWAS (Table S1).

### Assessment of population stratification

Multidimensional scaling analysis (Figure S1) and results of permutation tests for between-KD and control group identity-by-state (IBS) differences (*p* = 0.460) suggested that strong population stratification was absent. Furthermore, the variance inflation factor for genomic control, λ = 1.118, also indicated that no very strong stratification existed. The IBS sharing method implemented in PLINK showed no encrypted family relationships among cases and controls.

### GWAS and cross-platform validation

**Figure 1:**
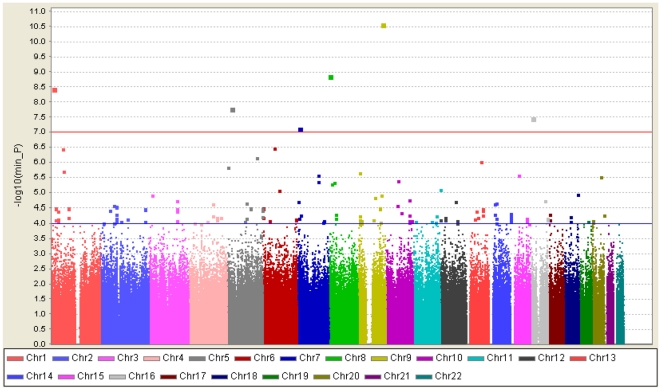
Graphical summary of KD GWAS in a Han Chinese population. KD association was determined for SNPs on the Affymetrix SNP6.0 chip. The y-axis represents the −log_10_ *p-*value, and the x-axis represents each of the 723,638 SNPs used in the primary scan of 250 KD cases and 446 controls.

The analysis was first performed with samples from 250 individuals with KD and 446 controls (Table S2). A total of 141 of the 723,638 SNPs were associated with KD (*p*<10^−4^) (Figure 1). Seventy-four of 141 SNPs with significant SNPs on the same LD block (the LD blocks were estimated based on Asian HapMap), good Affymetrix calling of clustering in both cases and controls, and located in (or within 500 kb) of known genes were chosen for cross-platform validation using Sequenom MassARRAY. Of these 74 SNPs, 57 that reached an overall 99% consistency and had association *p_best_-*values <10^−4^ with any genotype, allele, trend, dominant, or recessive models in both platforms were retained after cross-platform validation (Table S3).

### Replication of identified SNPs in a follow-up cohort

**Figure 2:**
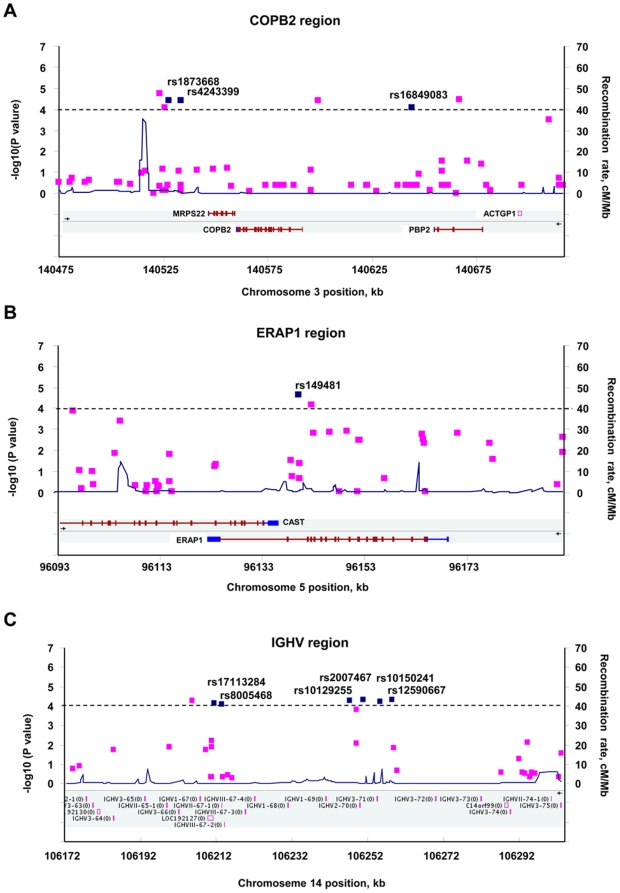
LD structure and association results for disease-association regions. The −log_10_ *p* values (left y-axis) for the best test from the primary scan were plotted as a function of genomic positions based on NCBI Build 36. The SNPs with the strongest signals in the joint analysis are denoted by blue diamonds. Estimated recombination rates (right y-axis) based on the Chinese HapMap population are plotted in blue across the region to reflect the local LD structure around the significant SNPs. Gene annotations were taken from NCBI.

We then genotyped an additional 208 cases and 366 controls for the 57 validated KD-associated SNPs. The results of this Stage 2 analysis and the joint analysis of 458 cases and 812 controls are listed in Table S3. Twelve of 57 SNPs had nominal *p*-values <0.05 in the replication cohort. Thirteen SNPs with the most significant association with KD in the joint analysis (*p_best_*<10^−4^) are listed in Table1 and highlighted in Table S3. Of these 13 SNPs, 10 in three regions with consecutive surrounding significant SNPs were selected; these are described below. The estimated recombination rates based on the Chinese HapMap and the association results from GWAS in the three regions are shown in Figure 2.

**Table 1:** SNPs showing the strongest association with KD in a Han Chinese population.

					Stage 1				Stage 2				Joint analysis (Stage 1+2)			
					(250 cases and 446 controls)				(208 cases and 366 controls)				(458 cases and 812 controls)			
SNP	Chr	Nearest gene(s)	Non-risk allele	Risk allele	RAF (KD)	RAF (Control)	OR (95% CI)	*p*-value (best)	RAF (KD)	RAF (Control)	OR (95% CI)	*p*-value (best)	RAF (KD)	RAF (Control)	OR (95% CI)	*p*-value (best)
rs1873668	3	MRPS22	C	A	0.05	0.02	3.549 (1.835–6.865)	3.68E-05	0.03	0.02	1.579 (0.754–3.303)	2.22E-01	0.04	0.02	2.501 (1.545–4.047)	9.52E-05
rs4243399	3	COPB2	A	G	0.05	0.02	3.541 (1.831–6.850)	3.82E-05	0.03	0.02	1.579 (0.754–3.303)	2.22E-01	0.04	0.02	2.494 (1.541–4.037)	9.93E-05
rs16849083	3	PBP2	G	A	0.05	0.02	3.564 (1.842–6.895)	3.46E-05	0.03	0.02	1.497 (0.705–3.178)	2.91E-01	0.04	0.02	2.837 (1.714–4.697)	2.19E-05
rs13128867	4	SLC7A11	C	T	0.33	0.23	1.618 (1.268–2.065)	7.42E-05	0.33	0.27	1.316 (1.010–1.716)	4.16E-02	0.33	0.25	1.469 (1.228–1.758)	2.23E-05
rs149481	5	ERAP1	G	T	0.83	0.73	1.825 (1.377–2.418)	2.25E-05	0.78	0.74	1.261 (0.949–1.676)	6.84E-02	0.80	0.73	1.506 (1.235–1.837)	4.61E-05
rs362794	7	RELN	A	G	0.11	0.04	2.537 (1.652–3.897)	4.41E-06	0.07	0.06	1.308 (0.808–2.119)	2.34E-01	0.09	0.05	1.900 (1.386–2.604)	3.03E-05
rs17113284	14	IGHV	T	C	0.42	0.33	1.441 (1.152–1.803)	7.22E-05	0.38	0.34	1.182 (0.926–1.509)	2.52E-02	0.40	0.34	1.314 (1.114–1.549)	1.08E-05
rs8005468	14	IGHV	C	T	0.42	0.33	1.449 (1.158–1.813)	7.98E-05	0.38	0.34	1.184 (0.928–1.511)	2.34E-02	0.40	0.34	1.321 (1.120–1.558)	8.93E-06
rs10129255	14	IGHV	G	A	0.42	0.33	1.462 (1.168–1.830)	5.17E-05	0.38	0.34	1.160 (0.910–1.480)	2.19E-02	0.40	0.34	1.315 (1.116–1.551)	6.77E-06
rs2007467	14	IGHV	G	A	0.42	0.33	1.479 (1.181–1.853)	4.66E-05	0.38	0.34	1.143 (0.896–1.458)	3.54E-02	0.40	0.34	1.307 (1.108–1.542)	1.28E-05
rs10150241	14	IGHV	T	A	0.42	0.33	1.447 (1.157–1.810)	5.73E-05	0.38	0.35	1.152 (0.902–1.470)	3.06E-02	0.40	0.34	1.304 (1.106–1.538)	1.08E-05
rs12590667	14	IGHV	G	A	0.42	0.33	1.456 (1.164–1.822)	4.81E-05	0.38	0.35	1.141 (0.893–1.457)	3.90E-02	0.40	0.34	1.303 (1.105–1.537)	1.27E-05
rs1568657	15	BTBD1	A	G	0.63	0.52	1.542 (1.228–1.936)	8.69E-05	0.58	0.53	1.261 (0.981–1.620)	1.80E-02	0.61	0.52	1.409 (1.191–1.667)	6.61E-06

Chr, chromosome; Risk allele, allele with higher frequency in cases compared to controls; RAF (KD) and RAF (control), risk allele frequencies in cases and controls, respectively; OR, odds ratio for risk allele; *p*-value (best), minimal *p*-value of the five association tests: genotype, allele, trend, dominant, and recessive. Stage 1 (genome scan) included 250 cases and 446 controls. Stage 2 (replication stage) included 208 cases and 366 controls. Alleles were indexed to the forward strand of NCBI Build 36.

The SNPs, rs1873668 (*p_best_* = 9.52×10^−5^), rs4243399 (*p_best_* = 9.93×10^−5^), and rs16849083 (*p_best_* = 9.93×10^−5^) are located in chromosome region 3q23 and were associated with KD in joint analysis (p*_best_*<10^−4^; Table 1). Their nearby SNPs, rs1873666 (*p_best_* = 0.000123), rs4894410 (*p_best_* = 0.000103), and rs16849065 (*p_best_* = 0.000243), also showed significant association in the joint analysis (Table S3). These SNPs were located to a linkage disequilibrium (LD) block containing genes encoding mitochondrial ribosomal protein S22 (MRPS22), coatomer protein complex beta-2 subunit (COPB2), and retinol-binding protein 2 (PBP2; Figures 2A and S2).

rs149481, with a *p_best_*-value of 4.61×10^−5^ in the joint analysis, is located in chromosome region 5 15q (Table 1) within the intronic region of the *ERAP1* (endoplasmic reticulum amino peptidase 1) gene (Figure 2B). The nearby SNP, rs27042, showed an association with KD in the joint analysis (*p_best_* = 0.000153; Table S3) and is also located in the same intronic region of the *ERAP1* gene (Figure 2B). ERAP1 plays a role in peptide trimming in the generation of most HLA (human leukocyte antigen) class I–binding peptides, and is also involved in regulating proinflammatory cytokine signaling through cleavage of cytokine cell surface receptors.

A cluster of variants was located in an LD block of chromosome region 14 q32.33 (Figure S2). This cluster included rs17113284 (*p_best_* = 1.08×10^−5^), rs8005468 (*p_best_* = 8.93×10^−6^), rs10129255 (*p_best_* = 1.14×10^−5^), rs2007467 (*p_best_* = 1.28×10^−5^), rs10150241 (*p_best_* = 1.08×10^−5^), and rs12590667 (*p_best_* = 1.27×10^−5^) (Tables 1 and S3). This region contains genes coding for immunoglobulin heavy chain variable regions (Figure 2C).

### Comparison of our results with previously identified candidate regions

Some genes with known functions or roles related to KD pathophysiology have been intensively studied for an association with KD using the candidate gene approach. Therefore, we examined the SNPs in these gene regions in our GWAS to identify their association with KD in the Han population. HLA-Bw22 (Bw54) has been associated with KD in a Japanese population [14], and a SNP located in the *HLA-E* gene was suggested to be associated with KD in the Han Chinese population [15]. Additionally, a SNP for the TNF-α 308A variant was shown to be associated with susceptibility to KD in a Chinese population [16], and a Taiwanese group reported that an insertion in the angiotensin I converting enzyme was associated with KD susceptibility [17]. More importantly, an association of the *ITPKC* (inositol 1,4,5-trisphosphate 3-kinase C) gene region with KD susceptibility was identified by linkage analysis in Japanese and US cohorts, and the encoded protein was shown to be involved in T-cell activation in an in vitro cell system [12]. Our results showed that none of the SNPs located in or near these gene regions had −log10 (*p_best_*) values greater than 4 in our GWAS. However, some SNPs located in *HLA-A*, *HLA-B*, *TNF*-α, and *ITPKC* regions did show a nominal association (P<0.01) in our GWAS (Figure S3).

SNPs located in eight regions with *p*-values <0.05 were previously identified in a combined analysis of an international cohort of Caucasian KD patients and in a family-based KD cohort [13]. Therefore, we further examined the SNPs from these gene regions that were included in our GWAS. Our results showed that none of these SNPs had −log10 (*p_best_*) values greater than 4. However, 10 and 42 SNPs in *NAALADL2* (N-acetylated alpha-linked acidic dipeptidase-like 2) and *CSMD1* (CUB and Sushi multiple domains) regions, respectively, showed a nominal association with KD (*p*<0.01) in our GWAS (Figure S4).

## Discussion

To the best of our knowledge, this study is the first report of a GWAS for KD conducted in a Han Chinese population. In this study, we identified novel candidates associated with KD susceptibility. The identification of these genes may provide new insights into the development of KD.

Three KD-associated SNPs—rs1873668, rs4243399, and rs16849083—are located in the same LD of chromosome 3q23 region, which contains three genes. The head-to-tail overlapping genes *MRPS22* and *COPB2* are located nearest to rs1873668 and rs4243399, whereas rs16849083 is located in the intronic region of *PBP2*. Mutations in MRPS22, a mitochondrial ribosomal protein, lead to antenatal mitochondrial disease, which presents with antenatal skin edema, hypotonia, cardiomyopathy, and tubulopathy [18]. PBP2 is thought to participate in the uptake and/or intracellular metabolism of vitamin A. The roles of NRPS22 and PBP2 have no obvious connection to the pathogenesis of KD. COPB2 constitutes the coat of nonclathrin-coated vesicles and is essential for Golgi budding and vesicular trafficking. A phosphoproteomic analysis of T cell receptor signaling, which identified proteins involved in receptor and membrane trafficking in endosomes and vesicles playing a role in T cell receptor signaling and critical for T cell activation [19]. The possibility of COPB2 involvement in T cell activation and KD pathogenesis will require further investigation.

We identified two KD-associated SNPs, rs149481 and rs27042, located in an intron of the *ERAP1* gene. ERAP1 plays a critical role in trimming peptides to the optimal length for HLA class I presentation [20] and cleaving cell surface receptors for proinflammatory cytokines. It is ubiquitously expressed in every tissue, and is expressed at higher levels in the trachea, thymus, and lymph nodes (based on a National Center for Biotechnology Information [NCBI] expressed sequence tags profile). From a functional perspective, ERAP1 represent an excellent biological candidate for KD pathogenesis. ERAP1 trimming of peptides for HLA class I presentation could play a critical role in initiating the immune response. Furthermore, ERAP1 cleaves cell surface receptors for the proinflammatory cytokines IL-1 [21], IL-6 [22], and TNF [23], thereby downregulating their signaling. Genetic variants that alter the functioning of ERAP1 could, therefore, have proinflammatory effects through this mechanism. In addition to their association with KD, ERAP1 polymorphisms have been found to be associated with ankylosing spondylitis, an HLA class I–mediated autoimmune disease, and enthesitis, a common inflammatory arthritis characterized by axial skeletal inflammation [24]. We did not identify any significant SNPs in the HLA region in our GWAS, suggesting that augmentation of proinflammatory cytokine signaling through ERAP1 is more likely involved in KD. However, the association between HLA and KD remains inconclusive. Some SNPs located in *HLA-A* and *HLA-B* regions did show a nominal association (*p*<0.01) in our study. Determining whether HLAs play a role in KD susceptibility will require further investigation in a larger sample size using a traditional HLA genotyping method. Clarifying a potential association between HLA and KD will also help to elucidate the involvement of an ERAP1 downstream mechanism in KD.

We found that a cluster of SNPs located in chromosome 14 immunoglobulin heavy chain variable regions exhibited the strongest association in the joint analysis. The heavy chain molecule is a major contributor to the generation of immunoglobulin diversity and specificity. Therefore, in addition to cell-mediated immunity, antibody-mediated immunity may be critical in KD pathogenesis. IgA plasma cells infiltrate inflamed tissues, including coronary arteries, in acute KD [25], [26], and oligoclonal KD antibodies bind to an antigen in acute KD-inflamed ciliated bronchial epithelium [27]. Specific immunoglobulins produced by particular heavy chain recombinants following a KD antigen trigger could play a role in KD pathogenesis. Identification of these specific antibodies could lead to the elucidation of disease-relevant mechanisms and potential treatments.

We also performed a haplotype analysis, identifying two haplotypes with *p*-values <10^−5^. The significance of these two haplotypes, however, was mainly contributed by single SNPs showing strong associations in single-point analyses. Also, the haplotype frequencies were less than 0.05. Accordingly, we could not exclude the possibility that the significant *p*-values and the small differences between the haplotype frequencies of cases and controls might have resulted from estimation errors, given the small sample sizes. Additional analyses with larger numbers of subjects are warranted.

The major limitation of this study is that the low sample size, which reflected the difficulties inherent in recruiting patients with such a rare disease, could only reach a statistical power of 0.78 to detect a disease allele with a frequency of 0.10 and a relative risk of 3.0, assuming a disease incidence of 0.01% under an additive model. Also, the small sample size is underpowered to detect very small effects. With the additional replication sample, the joint analysis marginally strengthened some of the SNPs in the GWAS. Many of the significantly associated SNPs in the discovery phase were either insignificant or of marginal significance in the replication phase. This raises the possibility of false positives, a problem common to all GWAS. Replication of the findings in large, well-powered independent samples is crucial if this problem is to be overcome, and will likely require a multicenter collaboration. Such an increase in the size of the study cohorts would also make it possible to detect smaller effects. In addition, we found a higher proportion of male KD patients than controls in our study because of the higher incidence of KD in males. Although it is unclear whether and how this might have affected the results, it could have led to some degree of bias. Several genes implicated in previous candidate gene studies were not shown to have −log10 (*p_best_*) values greater than 4 in our studies, probably due to difference in the relative importance of particular genetic variants in determining susceptibility to complex diseases among different ethnic groups or ethnicity-linked haplotype structures. However, some of the SNPs located in the candidate gene regions did show a KD association with *p*-values <0.01. Further replication with a larger sample size and different populations would be helpful in evaluating the involvement of these genes in the susceptibility of KD.

In summary, we conducted a GWAS for KD in a Han Chinese population. Ten SNPs located in three novel loci were found to be associated with KD in this population. The novel KD risk loci contained genes that have been implicated in T cell receptor signaling, proinflammatory cytokine regulation, as well as antibody-mediated immune responses. Our results suggest that antigen initiation, uncontrolled inflammation, and antibodies are involved in the pathogenesis of KD, findings that may provide new directions for future studies. In particular, further characterization of causative variants and elucidating their mechanisms of action may lead to the identification of novel diagnostic and therapeutic targets for KD.

## Materials and Methods

### Ethical statement

The study was approved by the Institutional Review Board and the Ethics Committee of the Institution Review Board of China Medical University Hospital, National Taiwan University Hospital, Changhua Christian Hospital, Taipei Veterans General Hospital, Kaohsiung and Linkou Chang Gung Memorial Hospital, Mackay Memorial Hospital, and Academia Sinica, Taiwan. Written informed consent was obtained from the subjects or their parents in accordance with institutional requirements and the Declaration of Helsinki principles.

### Study subjects and phenotype definition

Unrelated patients with KD (n = 250) were consecutively recruited from China Medical University Hospital in Taichung, Taiwan, in collaboration with the National Clinical Core (NCC) of Taiwan. There were 156 males (62.4%) and 94 females (37.6%) (Table S2), with an average age at onset of 1.76±1.61 years. The 208 KD patients for the follow-up validation study were recruited from China Medical University Hospital in Taichung, National Taiwan University Hospital in Taipei, Changhua Christian Hospital in Changhua, Taipei Veterans General Hospital in Taipei, and Chang Gung Memorial Hospital in Kaohsiung and Linkou, Taiwan. There were 142 males (68.3%) and 66 females (31.7%) in this second study group (Table S2). All patients were diagnosed according to criteria for KD [28], [29], including fever lasting 5 d or more and at least four of the following symptoms: (1) changes in extremities (e.g., erythema, edema, desquamation), (2) bilateral conjunctival injection, (3) polymorphous rash, (4) cervical lymphadenopathy, (5) changes in lips or oral cavity (e.g., pharyngeal erythema, dry/fissured or swollen lips, strawberry tongue). Only Han Chinese individuals, who account for 98% of Taiwanese residents, were considered for recruitment. The ethnic background was assigned based on self-report questionnaires. Members of the control groups (446 in the GWAS study and 366 in the follow-up study) were randomly selected from the Taiwan Han Chinese Cell and Genome Bank in Taiwan, as reported previously [30]. The prevalence of KD in the Taiwan population is less than 0.01%; hence, the controls were presumably disease free.

### Genotyping and quality control

Genomic DNA was extracted from patient blood using the Puregene DNA Isolation Kit (Gentra Systems, Minneapolis, MN, USA). Each individual was genotyped by the National Genotyping Center at Academia Sinica, Taipei, Taiwan using the Affymetrix Genome-Wide Human SNP Array 6.0, containing a total of 906,600 SNPs, according to the manufacturer's protocols. Genotype calls were made using the BRNNP algorithm implemented in Genotype Console, with default parameters suggested by the platform manufacturer. All sample call rates were greater than 0.95, and the average call rate of the sample was 0.993 (SD = 0.005). Genotype data was quality controlled by examining several summary statistics. First, the total call rate (successful call rate) and MAF of cases and controls was calculated for each SNP. SNPs were excluded for further analysis if one of following conditions occurred: (1) only one allele appeared in cases and controls; (2) the total call rate was less than 0.95; or (3) the total MAF was less than 0.05 and the total call rate was less than 0.99. SNPs that significantly departed from Hardy–Weinberg equilibrium proportions (*p*<10^−7^) were also excluded.

### Population stratification

Possible population stratification that could influence association analyses was detected using multidimensional scaling analysis implemented in PLINK (http://pngu.mgh.harvard.edu/purcell/plink [30]). We also estimated the variance inflation factor for genomic control.

### Genome-wide association analysis

GWAS was carried out to compare allele/genotype frequencies between cases and controls using genotype, allele type, and Cochran–Armitage trend tests, and tests considering dominant and recessive inheritance modes. After applying these five single-point methods, quantile–quantile (Q–Q) plots were used to examine *p*-value distributions (Figure S5). Two-point analyses were performed using a logistic regression model, regressing the affected status of two SNPs and their interaction. SNPs were coded as 0, 1, and 2 for the number of minor alleles and were treated as continuous variables.

### Validation and replication

The top SNPs (*p*<10^−4^) from the GWAS in the 250 cases and 446 controls were further validated using matrix-assisted laser desorption/ionization-time of flight (MALDI-TOF) mass spectrometry (Sequenom MassARRAY; Sequenom, San Diego, CA, USA). SNPs retained after cross-platform validation were then genotyped in an additional 208 cases and 366 controls for replication.

## Supporting Information

Figure S1 **Multidimensional scaling analysis plot.** (A) Multidimensional scaling (MDS) plot of the first two principal components estimated by PLINK, based on the genotype data of 100,000 SNPs with equal spacing across the human genome randomly selected from 723,638 high-quality SNPs in KD cases and controls with HapMap3 genotype data was presented. (B) The results of KD cases and controls shown separately. No population stratification between the 250 KD cases (red) and 446 controls (blue) was detected (*p*  =  0.460).
(TIFF)

Figure S2 **Linkage disequilibrium plot of disease-association regions.** The upper panel shows –log_10_ (*p*-values) of SNPs for the best test from the primary scan as a function of genomic positions for (A) COPB2, (B) ERAP1, (C) IGHV regions (based on NCBI Build 36). Also shown are the relative positions of genes that map to each region of association. The SNPs with the strongest signal in the joint analysis are denoted by red diamonds. In the lower panel are the estimated statistics of the square of the correlation coefficient (r^2^), derived from genotypes in our GWAS. The values indicate the LD relationship between each pair of SNPs shown within each diamond. Red diamonds without numbers represent D'  =  1.
(TIFF)

Figure S3 **Comparisons with previously identified KD susceptibility gene regions.** Comparison of results for KD susceptibility gene regions encoding (A) HLA-A, (B) HLA-B, and (C) TNF-α, reported in previous candidate gene studies, and (D) ITPKC identified in a genome-wide linkage study, to those in the current study. Triangles represent the −log_10_ of the minimal *p*-values for the SNPs in the gene region and within a ±200-kb region, based on the initial analysis of 250 KD cases and 446 controls. Also shown are the relative positions of genes that map to each region of association (based on NCBI Build 36). In the lower panel is the estimated square of the correlation coefficient (r^2^), derived from phase-2 genotypes in CHB and JPT in Haploview software (v4.1). The red diamonds indicate the LD relationship between each pair of SNPs as D'  =  1.
(TIFF)

Figure S4 **Comparisons to previous GWAS.** Comparison of the present results for KD susceptibility gene regions (A) NAALADL2 and (B) CSMD1 to those reported by previous GWAS. Squares represent the −log_10_ of the minimal *p*-values of the SNPs in the gene region, based on the initial analysis of 250 KD cases and 446 controls. Also shown are the relative positions of genes that map to each region of association (based on NCBI Build 36). In the lower panel is the estimated square of the correlation coefficient (r^2^), derived from phase-2 genotypes in CHB and JPT in Haploview software (v4.1). The values indicate the LD relationship between each pair of SNPs shown within each diamond. Red diamonds without numbers represent D'  =  1. The same SNPs genotyped in the previous GWAS and in our GWAS are denoted by red diamonds in the upper panel and a red rectangle in the lower panel.
(TIFF)

Figure S5 **Q–Q plot for the trend test.** Q–Q plots are shown for the trend test based on the 723,638 high-quality SNPs of the initial analysis with 250 cases and 446 controls. Blue lines represent the upper and lower boundaries of the 95% confidence interval bands. Black dots showing deviations from the line of equality indicate either that the theoretical distribution was incorrect, or that the sample was contained within values generated by a true association.
(TIFF)

Table S1 **Quality control of participant data.**
(PDF)

Table S2 **Demographic and clinical characteristics of participants in the GWAS and replication study.**
(PDF)

Table S3 **Association results and concordance rates for validated, significantly associated SNPs in the initial cohort, and association results from the replication study.**
(PDF)
