# Supplementary figures and images for: Identification of Novel Susceptibility Loci for Kawasaki Disease in a Han Chinese Population by a Genome-Wide Association Study
Source: PLoS One. 2011 Feb 4;6(2):e16853. doi: 10.1371/journal.pone.0016853 (PMC3033903; doi:10.1371/journal.pone.0016853)

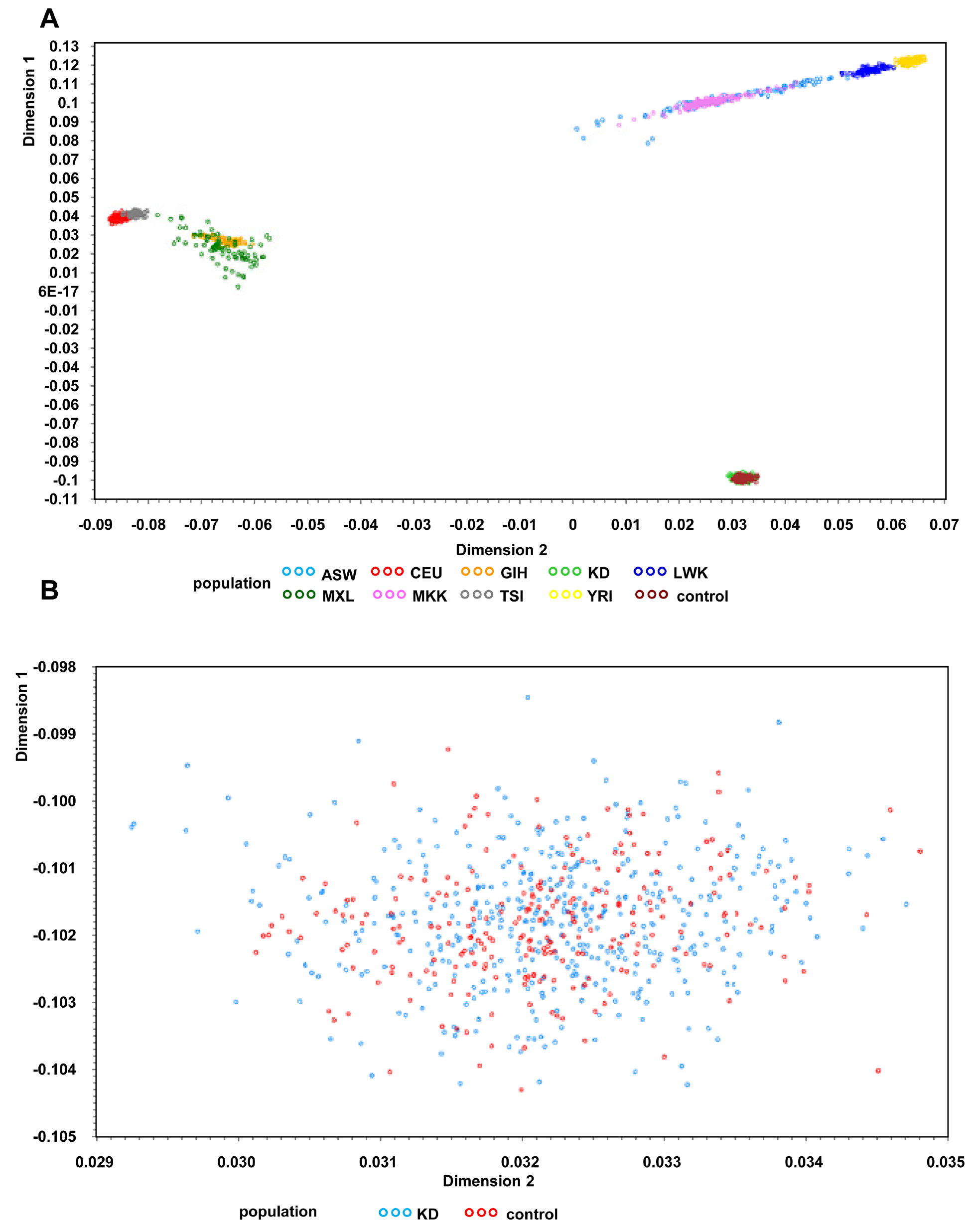

Supplement: Figure S1 — Multidimensional scaling analysis plot. (A) Multidimensional scaling (MDS) plot of the first two principal components estimated by PLINK, based on the genotype data of 100,000 SNPs with equal spacing across the human genome randomly selected from 723,638 high-quality SNPs in KD cases and controls with HapMap3 genotype data was presented. (B) The results of KD cases and controls shown separately. No population stratification between the 250 KD cases (red) and 446 controls (blue) was detected (p = 0.460). (TIFF) [file pone.0016853.s001.tiff]

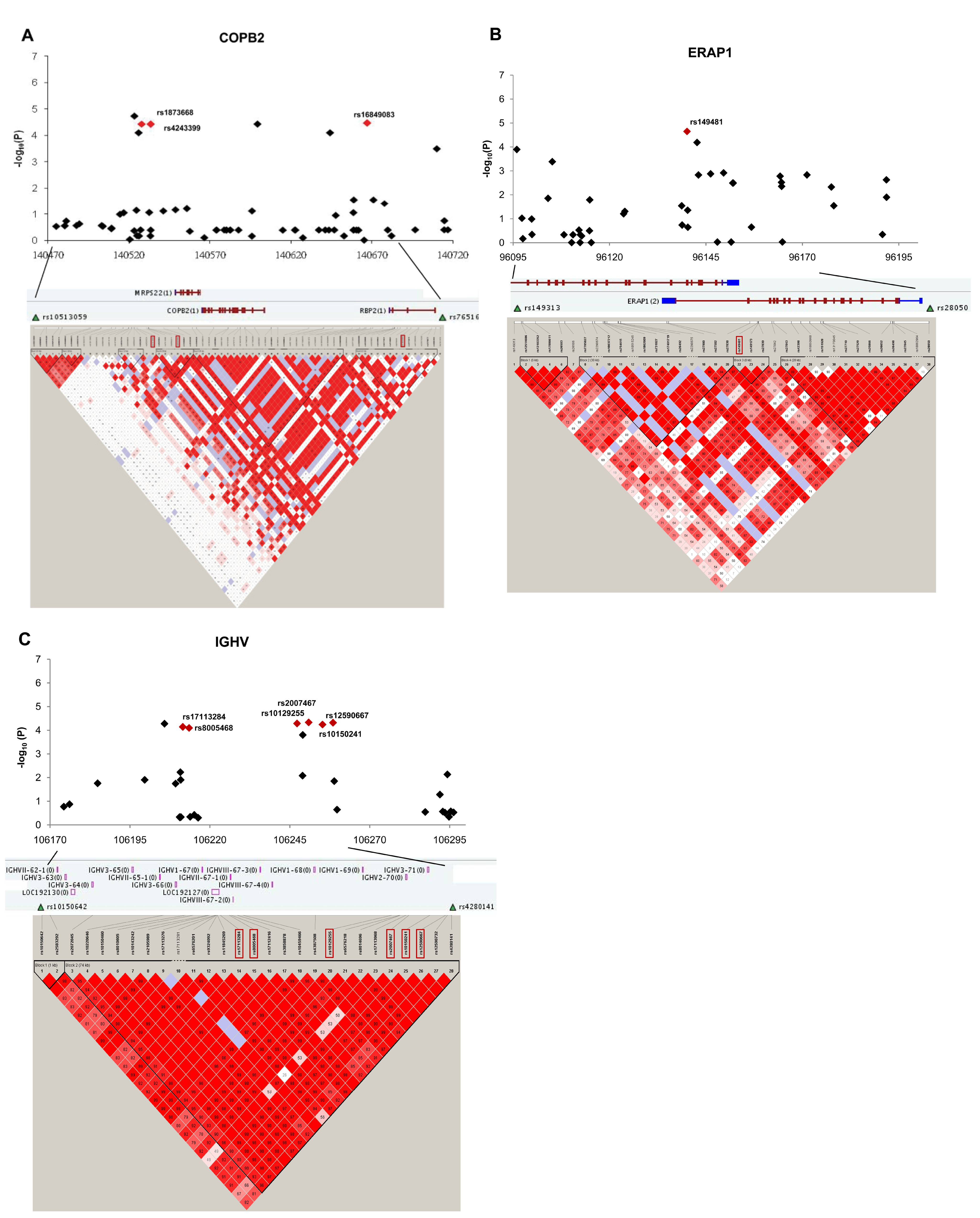

Supplement: Figure S2 — Linkage disequilibrium plot of disease-association regions. The upper panel shows –log10 (p-values) of SNPs for the best test from the primary scan as a function of genomic positions for (A) COPB2, (B) ERAP1, (C) IGHV regions (based on NCBI Build 36). Also shown are the relative positions of genes that map to each region of association. The SNPs with the strongest signal in the joint analysis are denoted by red diamonds. In the lower panel are the estimated statistics of the square of the correlation coefficient (r2), derived from genotypes in our GWAS. The values indicate the LD relationship between each pair of SNPs shown within each diamond. Red diamonds without numbers represent D' = 1. (TIFF) [file pone.0016853.s002.tiff]

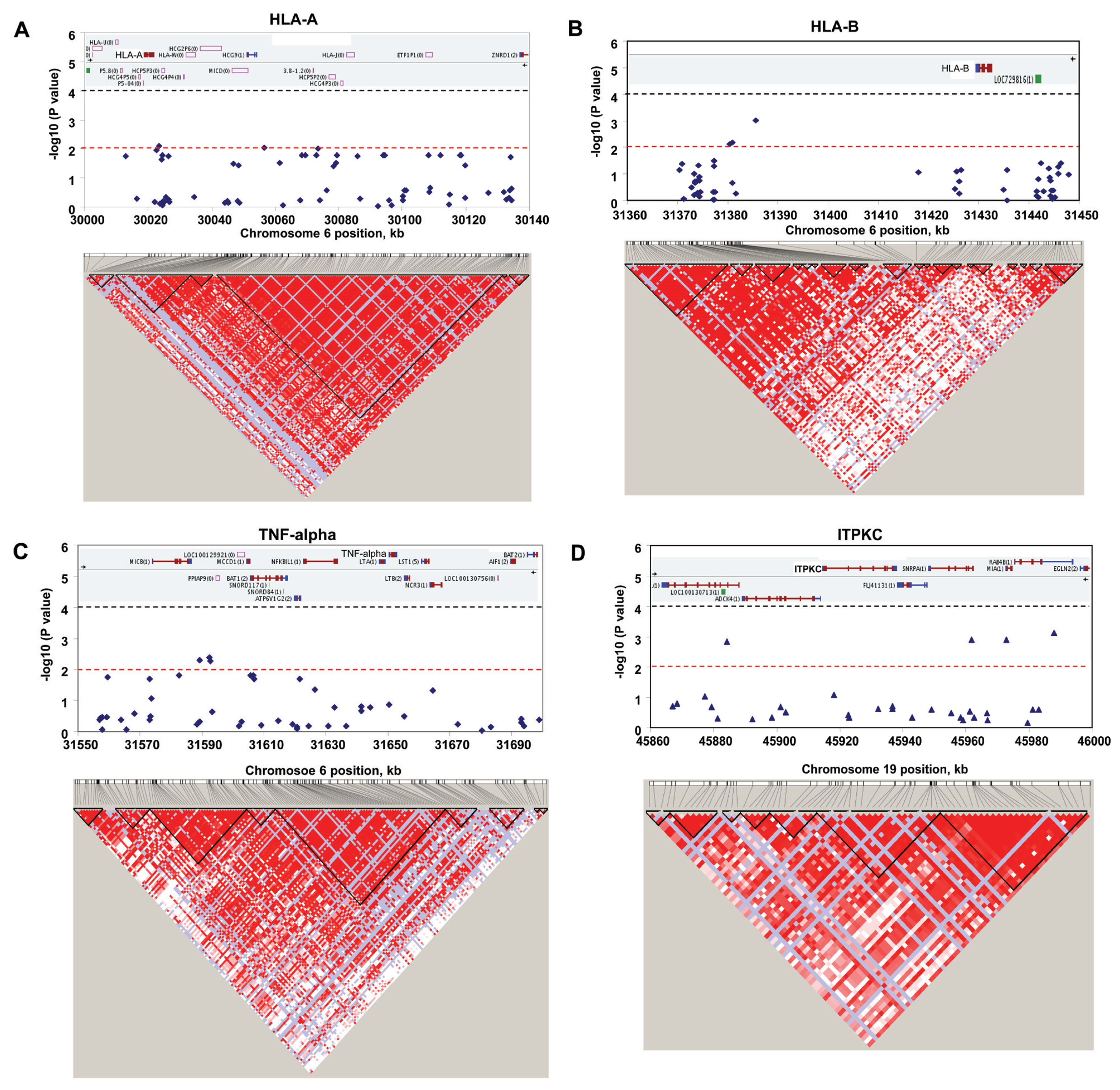

Supplement: Figure S3 — Comparisons with previously identified KD susceptibility gene regions. Comparison of results for KD susceptibility gene regions encoding (A) HLA-A, (B) HLA-B, and (C) TNF-α, reported in previous candidate gene studies, and (D) ITPKC identified in a genome-wide linkage study, to those in the current study. Triangles represent the −log10 of the minimal p-values for the SNPs in the gene region and within a ±200-kb region, based on the initial analysis of 250 KD cases and 446 controls. Also shown are the relative positions of genes that map to each region of association (based on NCBI Build 36). In the lower panel is the estimated square of the correlation coefficient (r2), derived from phase-2 genotypes in CHB and JPT in Haploview software (v4.1). The red diamonds indicate the LD relationship between each pair of SNPs as D' = 1. (TIFF) [file pone.0016853.s003.tiff]

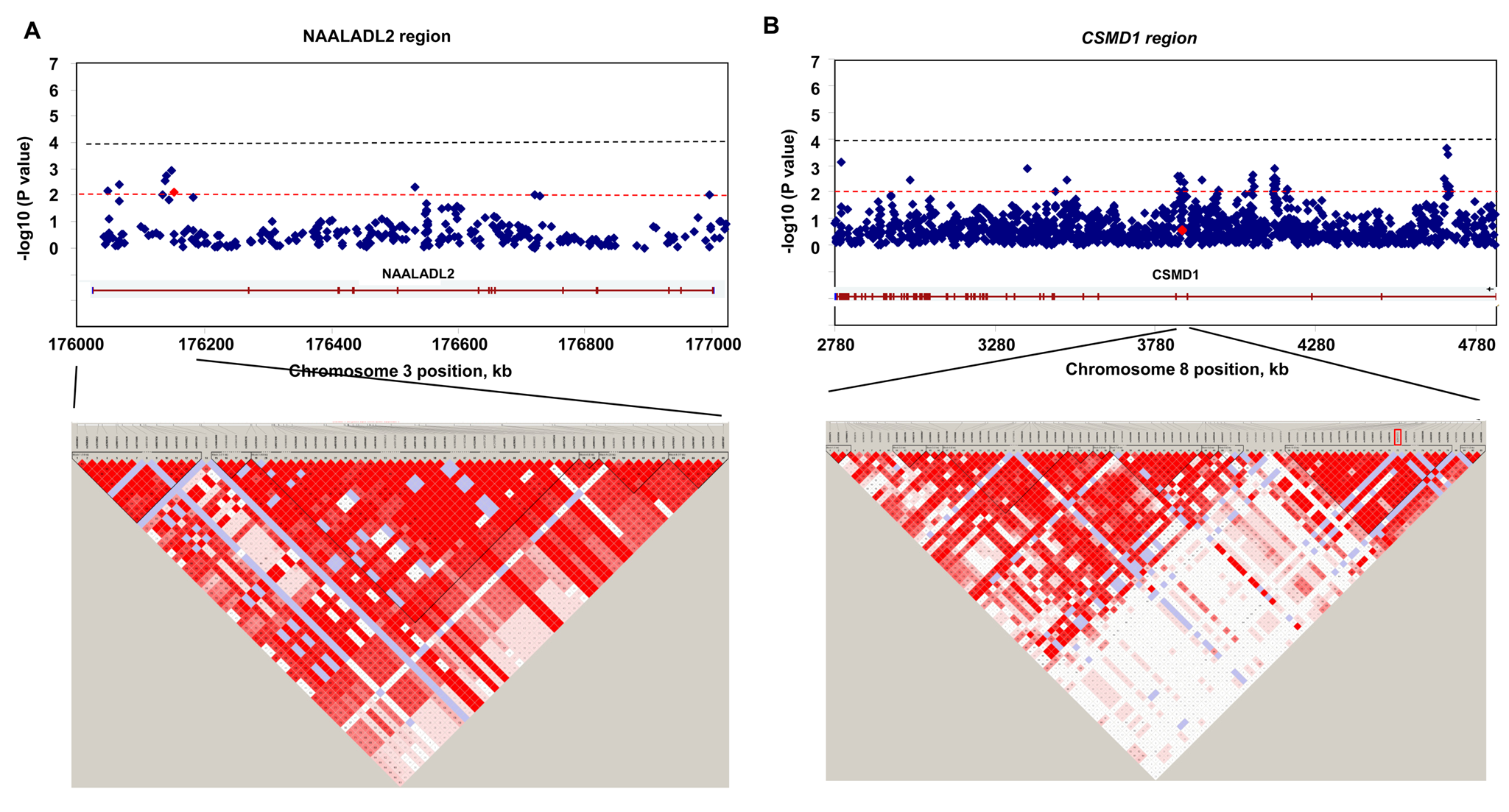

Supplement: Figure S4 — Comparisons to previous GWAS. Comparison of the present results for KD susceptibility gene regions (A) NAALADL2 and (B) CSMD1 to those reported by previous GWAS. Squares represent the −log10 of the minimal p-values of the SNPs in the gene region, based on the initial analysis of 250 KD cases and 446 controls. Also shown are the relative positions of genes that map to each region of association (based on NCBI Build 36). In the lower panel is the estimated square of the correlation coefficient (r2), derived from phase-2 genotypes in CHB and JPT in Haploview software (v4.1). The values indicate the LD relationship between each pair of SNPs shown within each diamond. Red diamonds without numbers represent D' = 1. The same SNPs genotyped in the previous GWAS and in our GWAS are denoted by red diamonds in the upper panel and a red rectangle in the lower panel. (TIFF) [file pone.0016853.s004.tiff]

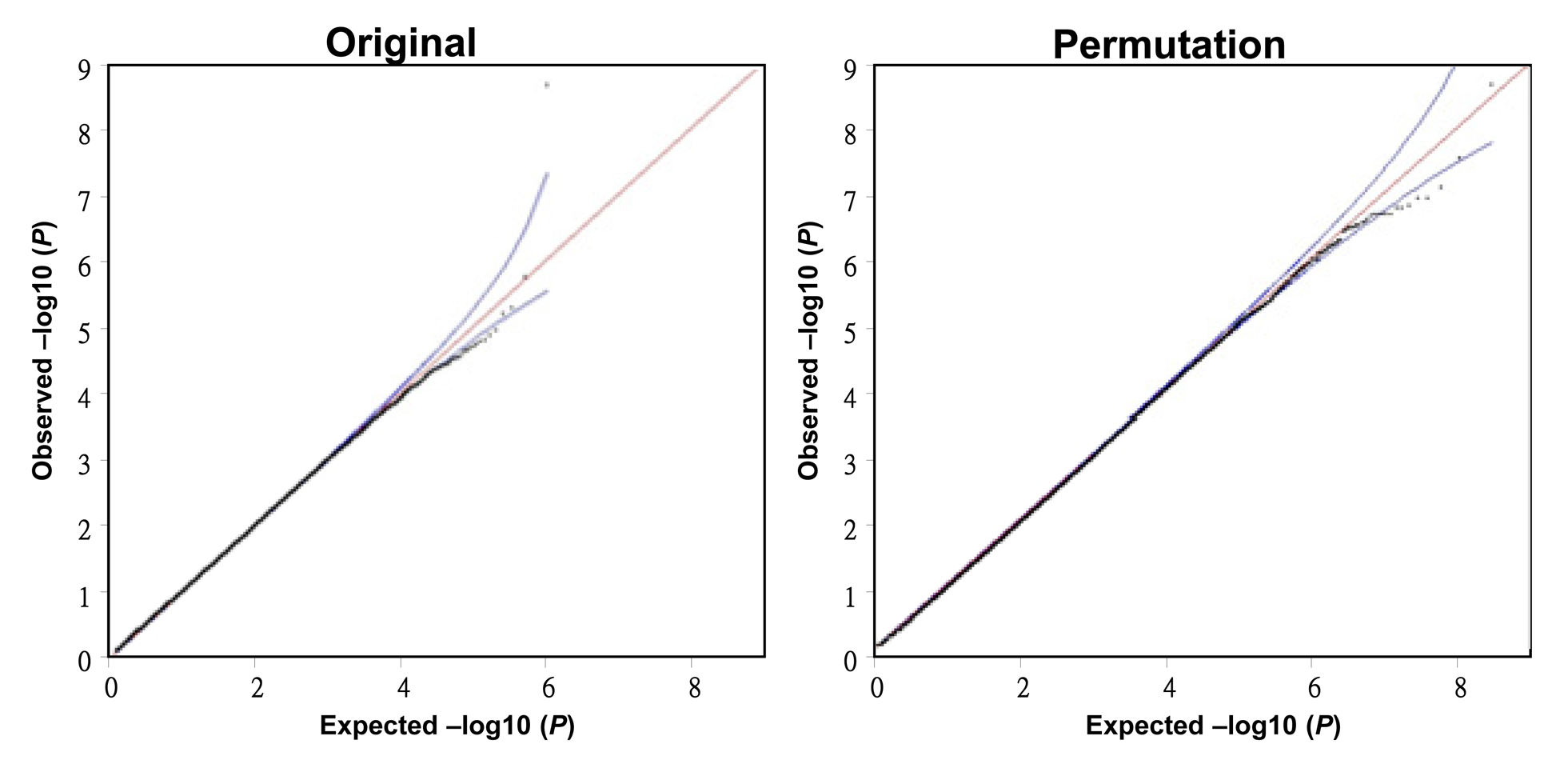

Supplement: Figure S5 — Q–Q plot for the trend test. Q–Q plots are shown for the trend test based on the 723,638 high-quality SNPs of the initial analysis with 250 cases and 446 controls. Blue lines represent the upper and lower boundaries of the 95% confidence interval bands. Black dots showing deviations from the line of equality indicate either that the theoretical distribution was incorrect, or that the sample was contained within values generated by a true association. (TIFF) [file pone.0016853.s005.tiff]
