# Supplementary material for: Identification of Novel Susceptibility Loci for Kawasaki Disease in a Han Chinese Population by a Genome-Wide Association Study
Source: PLoS One. 2011 Feb 4;6(2):e16853. doi: 10.1371/journal.pone.0016853 (PMC3033903; doi:10.1371/journal.pone.0016853)
Supplement: Table S1 — Quality control of participant data. (PDF) [file pone.0016853.s006.pdf]

Table S1. Quality control of participant data.

Number at start of QC	906,545 SNPs
Number dropped during exclusion step:	
Non-polymorphic in cases & controls	96,955 SNPs
Overall call rate < 95%	27,779 SNPs
Overall MAF < 5% & total call rate < 99%	30,080 SNPs
SNPs with HWE for controls ($p < 10^{-7}$) & no non-polymorphic in controls	28,093 SNPs
Number at end of QC	723,638 SNPs
