# Supplementary material for: Identification of Novel Susceptibility Loci for Kawasaki Disease in a Han Chinese Population by a Genome-Wide Association Study
Source: PLoS One. 2011 Feb 4;6(2):e16853. doi: 10.1371/journal.pone.0016853 (PMC3033903; doi:10.1371/journal.pone.0016853)
Supplement: Table S2 — Demographic and clinical characteristics of participants in the GWAS and replication study. (PDF) [file pone.0016853.s007.pdf]

Table S2. Demographic and clinical characteristics of participants in the GWAS and replication study.

Characteristics	Genome-Wide Association Cohort	Follow-up Cohort
Cases (% male)	250 (62.4%)	208 (68.3%)
Control (% male)	446 (52.9%)	366 (41.3%)
Coronary artery abnormality (%)	88/250 (35.2%)	52/208 (25%)
(% male)	65/88 (73.86%)	35/52 (67.31%)
