# Supplementary material for: Identification of Novel Susceptibility Loci for Kawasaki Disease in a Han Chinese Population by a Genome-Wide Association Study
Source: PLoS One. 2011 Feb 4;6(2):e16853. doi: 10.1371/journal.pone.0016853 (PMC3033903; doi:10.1371/journal.pone.0016853)
Supplement: Table S3 — Association results and concordance rates for validated, significantly associated SNPs in the initial cohort, and association results from the replication study. (PDF) [file pone.0016853.s008.pdf]

Table S3. Association results and concordance rates for validated, significantly associated SNPs in the initial cohort, and association results from the replication study.

Chr	SNP	Cytoband	Allele	concordance rates	pbest Stage 1	Stage 2	Join
1	rs12751986	p36.12	AG	99.67	3.24E-05	3.82E-02	3.05E-03
1	rs3117048	p36.12	CT	100	7.59E-05	3.39E-01	7.25E-04
1	rs771401	p34.3	AG	100	7.23E-05	2.85E-01	1.31E-03
1	rs880017	p34.3	AG	100	8.71E-05	1.42E-01	2.07E-04
1	rs4142781	p31.1	CT	100	3.22E-05	1.87E-01	6.91E-03
2	rs13391405	p14	CT	100	9.50E-05	4.10E-02	1.07E-01
2	rs1375373	p14	CG	99.84	2.60E-05	1.25E-01	1.00E-01
2	rs560737	p12	AG	100	2.79E-05	1.46E-01	6.65E-03
2	rs410532	p12	AG	99.51	5.05E-05	1.28E-01	2.72E-02
2	rs53915	p12	AG	99.84	6.45E-05	1.28E-01	1.89E-02
2	rs7606753	q35	AG	100	8.54E-05	5.17E-02	2.16E-04
3	rs1873666	q23	CT	99.84	1.84E-05	3.70E-01	1.23E-04
3	rs4894410	q23	CG	100	7.90E-05	1.87E-01	1.03E-04
3	rs1873668	q23	AC	100	3.68E-05	2.22E-01	9.52E-05
3	rs4243399	q23	AG	100	3.82E-05	2.22E-01	9.93E-05
3	rs16849065	q23	AG	99.84	7.90E-05	3.23E-01	2.43E-04
3	rs16849083	q23	AG	99.84	3.46E-05	3.17E-01	2.19E-05
4	rs4833167	q27	GT	100	2.42E-05	1.10E-01	2.94E-04
4	rs13128867	q28.3	CT	99.84	7.42E-05	4.16E-02	2.23E-05
4	rs4863532	q28.3	AG	99.84	6.09E-05	7.62E-01	5.01E-02
5	rs149481	q15	GT	100	2.25E-05	2.72E-01	4.61E-05
5	rs27042	q15	AG	100	6.53E-05	4.26E-02	1.53E-04
5	rs678591	q35.2	GT	99.84	6.04E-05	5.84E-01	6.40E-04
5	rs17601481	q35.2	CT	99.84	3.64E-05	6.57E-01	2.95E-04
5	rs6875659	q35.2	AG	100	3.46E-05	1.88E-01	2.71E-02
6	rs238050	p25.3	CT	100	6.15E-05	1.76E-02	2.83E-03
6	rs9365775	q27	AG	99.84	7.63E-05	3.30E-01	3.27E-03
6	rs1511085	q27	CT	100	7.11E-05	4.19E-01	3.13E-03
7	rs362794	q22.1	AG	100	4.41E-06	3.05E-01	3.03E-05

9	rs6477140	p24.1	CT	100	5.49E-05	3.34E-01	3.42E-04
9	rs303751	p22.3	CT	99.52	9.80E-05	7.74E-02	1.35E-03
9	rs913417	p22.3	AG	100	7.85E-05	2.85E-01	4.61E-04
9	rs1464666	q31.1	AC	99.67	3.19E-05	4.02E-01	1.01E-02
10	rs657497	q25.3	AG	99.52	1.77E-05	3.84E-01	1.69E-04
10	rs2616657	q25.3	AG	99.5	8.98E-05	3.00E-01	3.28E-03
10	rs642602	q25.3	AG	99.84	5.12E-05	2.97E-01	6.78E-04
12	rs11048538	p11.23	CT	100	6.27E-05	6.96E-01	1.52E-03
12	rs11048543	p11.23	AG	100	6.97E-05	6.96E-01	1.79E-03
12	rs11104677	q21.32	AG	100	7.82E-05	5.43E-01	9.61E-03
12	rs12423537	q21.32	AC	100	9.79E-05	4.86E-01	1.35E-03
13	rs9318846	q31.1	CT	100	3.57E-05	3.60E-01	9.76E-04
13	rs9318847	q31.1	AT	100	3.44E-05	3.60E-01	9.77E-04
13	rs9531328	q31.1	AG	100	3.57E-05	3.60E-01	9.76E-04
14	rs848035	q13.3	CT	99.84	9.50E-05	1.00E-01	3.77E-03
14	rs848034	q13.3	CT	94.71	6.67E-05	1.76E-01	4.25E-03
14	rs17113284	q32.33	CT	100	7.22E-05	2.52E-02	1.08E-05
14	rs8005468	q32.33	CT	99.84	7.98E-05	2.34E-02	8.93E-06
14	rs10129255	q32.33	AG	99.84	5.17E-05	2.19E-02	1.14E-05
14	rs2007467	q32.33	AG	99.84	4.66E-05	3.54E-02	1.28E-05
14	rs10150241	q32.33	AT	99.84	5.73E-05	3.06E-02	1.08E-05
14	rs12590667	q32.33	AG	99.52	4.81E-05	3.90E-02	1.27E-05
15	rs11259948	q25.2	CG	99.84	6.50E-05	1.13E-01	1.22E-02
15	rs1568657	q25.2	AG	99.84	8.69E-05	3.04E-02	6.61E-06
17	rs11655647	p13.1	CT	100	4.82E-05	3.47E-01	1.13E-02
18	rs6507234	q12.2	AG	100	6.05E-05	4.97E-01	4.23E-03
19	rs4390710	q13.2	CT	100	8.73E-05	1.87E-01	9.56E-03
19	rs2353678	q13.2	CG	100	8.73E-05	1.24E-01	9.56E-03

P_{best}-values indicate the minimal *p*-value of the five association tests: genotype, allele, trend, dominant, and recessive.

The 13 SNPs with association *p*-values less than10^{-4} in the joint analysis are highlighted in gray.
